# Cross-cultural validation of the motivation to change lifestyle and health behaviours for dementia risk reduction scale in the Dutch general population

**DOI:** 10.1186/s12889-020-08737-y

**Published:** 2020-05-27

**Authors:** Tessa Joxhorst, Joyce Vrijsen, Jacobien Niebuur, Nynke Smidt

**Affiliations:** Department of Epidemiology, University of Groningen, University Medical Centre Groningen, Hanzeplein 1, FA40, PO Box 30 001, 9700 RB Groningen, The Netherlands

**Keywords:** Dementia, Health behavior, Risk reduction behavior, Lifestyle, Cross-cultural validation, Validation study

## Abstract

**Background:**

This study aimed to translate and validate the Motivation to Change Lifestyle and Health Behaviours for Dementia Risk Reduction (MCLHB-DRR) scale in the Dutch general population.

**Methods:**

A random sample of Dutch residents aged between 30 and 80 years old were invited to complete an online questionnaire including the translated MCLHB-DRR scale. Exploratory and confirmatory factor analyses (EFA and CFA) were conducted to assess construct validity. Cronbach’s alpha was calculated to assess internal consistency.

**Results:**

Six hundred eighteen participants completed the questionnaire. EFA and Cronbach’s alpha showed that four items were candidate for deletion. CFA confirmed that deleting these items led to an excellent fit (RMSEA = 0.043, CFI = 0.960, TLI = 0.951, χ^2^/df = 2.130). Cronbach’s alpha ranged from 0.69 to 0.93, indicating good internal consistency.

**Conclusion:**

The current study demonstrated that the Dutch MCLHB-DRR scale is a valid scale for assessing health beliefs and attitudes towards dementia risk reduction among Dutch adults aged between 30 and 80 years old.

## Background

Dementia is a major public health concern for society. The prevalence of dementia increases rapidly, from 50 million cases worldwide in 2019 to an estimated 152 million cases in 2050 [1]. In the Netherlands dementia was the leading cause of death in 2018 [2]. The national expenses for dementia were 9,1 billion euros in 2017, which accounted for 10.3% of the total Dutch health care expenses [3]. Also the number of patients with dementia increases rapidly in the Netherlands, which is estimated to rise from 280,000 cases in 2018 to more than 520,000 cases in 2040 [4]. The increasing number of dementia patients carries a high socioeconomic burden for society, because of the associated rising health care costs and the burdensome effects of the disease on patients, their families and caregivers [5]. The World Health Organization (WHO) highlights dementia as a public health priority and advocates for action to decrease its social and economic burden [6].

The increase in the number of dementia patients is mainly attributable to population ageing, since age is the most important risk factor for dementia [7, 8]. In addition to non-modifiable risk factors for dementia like age and genetics, several studies suggested potential modifiable risk factors that are associated with dementia and in particular Alzheimer’s disease and vascular dementia [9–12]. Recently, the evidence for these potential modifiable risk factors for dementia was summarized by Livingston et al. (2017) [12]. They found that 35% of all dementia cases worldwide are attributable to nine modifiable risk factors and recommended to start interventions including more childhood education, promotion of exercise, reduction of smoking, maintaining social engagement and management of hypertension, diabetes, obesity, depression and hearing loss. It is estimated that these interventions might delay or prevent a third of all dementia cases [12]. Therefore, most of the aforementioned interventions are also included in the WHO Guidelines on risk reduction of cognitive decline and dementia [13].

Currently, there is no cure for dementia, so prevention of dementia is the key in fighting this disease. A diversity of multi-domain lifestyle interventions was conducted in elderly and people at risk for dementia in order to decrease the risk of developing dementia, including the Finnish Geriatric Intervention Study to Prevent Cognitive Impairment and Disability (FINGER) study, the Multi-domain Alzheimer Preventive Trial (MAPT) study, the Prevention of Dementia by Intensive Vascular care (preDIVA) study and the Healthy Ageing Through Internet Counselling in the Elderly (HATICE) trial [14–17]. The aforementioned studies showed some evidence for effectiveness of a multi-domain approach to prevent elderly from cognitive decline, but further research is needed [18–20]. Although health behavioural change is crucial for dementia risk reduction, changing behaviour is complex and many factors are related to the chances for successfully altering behaviour according to different social cognitive theories and models [21–25]. Measuring beliefs and attitudes towards lifestyle adaptations for dementia risk reduction may help to predict a person’s willingness to change lifestyle and behaviour aiming to reduce one’s risk of developing dementia.

The Motivation to Change Lifestyle and Health Behaviours for Dementia Risk Reduction (MCLHB-DRR) scale was developed in Australia and measures the beliefs and attitudes towards dementia and dementia risk reduction [26]. The MCLHB-DRR scale was based on the Health Belief Model (HBM), since the HBM was believed to be the best-suited social cognitive model for dementia risk reduction [26]. The HBM suggests that engagement in health-promoting behaviour is defined by a person’s subjective risk assessment of getting a condition and how serious this condition and its consequences are, the perceived benefits and barriers of performing this behaviour, a stimulus to trigger this behaviour, the desire to achieve an outcome, and the confidence in one’s ability to take action [27]. The MCLHB-DRR scale consists of 27 items and includes all seven subscales of the HBM: perceived susceptibility, perceived severity, perceived benefits, perceived barriers, cues to action, general health motivation and self-efficacy. The MCLHB-DRR scale is considered to be valid and reliable in Australians aged 50 years and older [26]. A Turkish version of the MCLHB-DRR scale has been cross-culturally validated and turned out to be a valid and reliable tool in individuals aged 40 years and older [28]. To the best of our knowledge, the MCLHB-DRR scale has not been cross-culturally validated in any other languages or countries.

There is currently no instrument available to measure attitudes and beliefs towards lifestyle and health behavioural changes for dementia risk reduction in the Netherlands. The MCLHB-DRR scale could be used to measure the attitudes and beliefs towards lifestyle adaptations for dementia risk reduction in the Dutch population. This induces the opportunity to use this scale in developing tailored interventions or education programs focused on lifestyle adjustments for dementia risk reduction. Therefore, the aim of the current study was to translate and validate the MCLHB-DRR scale in the Dutch general population aged between 30 and 80 years old.

## Methods

### Study design and participants

MCLHB-DRR data were collected among a random sample of residents of the municipality of Groningen aged between 30 and 80 years old. Participants between 30 years and 80 years old were included since health behaviour change for dementia risk reduction is important during midlife, but also in later life [12]. The prevalence of dementia in the municipality of Groningen is estimated at 1100 cases in 2020 [29]. The percentage of residents with dementia in the municipality of Groningen is generally lower compared to other municipalities, probably due to a lower average age of the residents [30]. Although healthy ageing is an important mission of the municipality of Groningen, to the best of our knowledge, dementia friendly initiatives or public health campaigns for dementia risk reduction do not exist yet. From the 101,518 residents of the municipality of Groningen, 4500 residents stratified for age (30–39, 40–49, 50–59, 60–69 and 70–80 years old) and gender (male, female) were randomly selected by a staff member of the municipality of Groningen, taken a response rate of 12% into account. This staff member was not involved in the data collection nor data analyses of this study. The selected 4500 residents were invited by letter to participate in an online survey about ‘Lifestyle and dementia’. Potential participants had access to the online survey using the web address which was mentioned in the letter. The translated MCLHB-DRR scale was the last part of this survey. The survey was built in Survey Monkey (SurveyMonkey Inc., San Mateo, California, VS). In order to increase the response rate, five vouchers of 20 Euros were raffled among the participants. Furthermore, participants were offered to receive the survey results on population level if they would finish the complete survey.

A pilot study was conducted to test the final version of the online survey ‘Lifestyle and dementia’. A total of 25 people aged 30 to 80 years who were living outside the municipality of Groningen participated in the pilot study. They were recruited within the network of the research team members. Results of the pilot study did not lead to any changes in the final Dutch version of the MCLHB-DRR scale.

### Questionnaire

The MCLHB-DRR scale consists of 27 items covering seven subscales: perceived susceptibility (4 items), perceived severity (5 items), perceived benefits (4 items), perceived barriers (4 items), cues to action (4 items), general health motivation (4 items) and self-efficacy (2 items). Items are answered on a 5 point Likert-scale, ranging from ‘strongly disagree’ (score = 1) to ‘strongly agree’ (score = 5) [26].

### Scale translation

For the translation of the MCLHB-DRR scale, we used the method of Beaton et al. (2000) [31]. Briefly, the MCLHB-DRR scale was translated into Dutch by three native Dutch translators, independently. Two of these translators were familiar with the concepts being examined in the questionnaire (the so-called informed translators). The third translator was not familiar with the content or concepts of the questionnaire (uninformed translator). All items, instructions and the response options of the questionnaire were translated.

Subsequently, the three translated versions were synthesized to one Dutch version by the informed translators. The discrepancies between the three translated versions were discussed between the informed translators, taking the original questionnaire into account.

Secondly, the synthesized Dutch version of the questionnaire was translated back into English by two independent native English speakers (uninformed translators). Both translators were not involved in the translation of the questionnaire from English to Dutch and were blinded to the original version of the questionnaire.

Afterwards all versions of the questionnaire, including the original version, the three translated versions, the synthesized Dutch version, the two back translations and all written reports about the decisions being made during the translation process were discussed by the informed translators. Special attention was paid to achieve semantic, idiomatic, experiential and conceptual equivalence between the source and target version of the questionnaire. After a comprehensive review of all versions of the questionnaire, consensus about the pre-final version of the questionnaire was reached.

Finally, the two back translations were combined in the best possible way and this version was send to the developers of the original scale [26] to check whether the meaning of the translated items was equivalent to the meaning of the original items. Their feedback was discussed, resulting in a small change in the translation of item 20 and item 25. Afterwards, the Dutch final version of the MCLHB-DRR scale was established.

### Statistical analysis

First, study population characteristics and characteristics of the MCLHB-DRR scale were calculated using descriptive statistics. Second, exploratory factor analysis (EFA) was performed. Maximum Likelihood estimation or Principal Axis Factoring was used depending on whether the data was roughly normally distributed or non-normally distributed, respectively. Oblique rotation was used as rotation method (delta (0)), which is taking into account correlations among factors. If the correlations between all factors were below 0.32, we changed to Varimax rotation [32]. Items that did not have a correlation of 0.20 or higher with any of the other items were deleted immediately. Items with a high correlation (> 0.70) with any of the other items, were considered carefully. Items with a factor loading below 0.30 on any of the factors were deleted immediately. Deletion of an item was considered if the item did not load sufficiently on one of the factors (< 0.50) or if an item had a cross-loading greater than 0.30 [33].

Internal consistency of the subscales was evaluated by item-total correlations and Cronbach’s alpha. Deletion of an item was considered when the item-total correlation of an item was below 0.30 [33]. Cronbach’s alpha values of 0.70 or higher were considered acceptable [34].

In addition, confirmatory factor analysis (CFA) was conducted. The following fit indices and their required levels were used to verify construct validity of the MCLHB-DRR scale: Root Mean Squared Error of Approximation (RMSEA) < 0.08 (moderate) and < 0.05 (excellent), Comparative Fit Index (CFI) and Tucker-Lewis Index (TLI) > 0.90 (moderate) and > 0.95 (excellent) and χ^2^/df < 3.0 [35, 36].

EFA was performed using IBM SPSS Statistics software version 23 (SPSS Inc., Chicago, IL, USA). CFA was analysed using Stata version 13 (StataCorp. 2013. Stata Statistical Software: Release 13. College Station, TX: StataCorp LP.). Participants who did not complete the whole MCLHB-DRR scale were excluded from data analysis.

### Ethics

This study was approved by the Medical Ethics Review Board of the UMCG. All participants provided informed consent.

## Results

### Participant recruitment

From the 4500 selected potential participants, 621 participants completed the survey, which resulted in a response rate of 14%. The data of the ‘cues to action’ subscale of three participants were missing. These participants were excluded, leaving a total of 618 participants for data analysis.

### Characteristics of the study population

The characteristics of the study population (*n* = 618) are presented in Table 1. The mean age (standard deviation (SD)) of the participants was 57.3 (13.5) years. More than half of the participants were female (54%) and were married or had a registered partnership (54%). Most participants completed tertiary education (59%), followed by upper secondary education (24%), lower secondary education (14%) and elementary education (2%). About 58% of the participants were employed. The percentage of participants having a relative with dementia or a non-relative with dementia was 45% and 21%, respectively. The response rate within the different strata was lower in the younger age categories (13% aged between 30 and 40, 12% aged between 40 and 50) compared to the older age categories (20% aged between 50 and 60, 30% aged between 60 and 70 and 22% aged between 70 and 80). However, the distribution of males and females was equally distributed within the age categories, except for the youngest age category aged 30 to 40 (female 67%; male 33%).

**Table 1:** Study population characteristics

Characteristic	All participants (***N*** = 618)^**a**^
Age, years (mean ± SD)	57.3 ± 13.5
Gender (% male)	281 (46%)
Marital status	
Married/registered partnership	336 (54%)
Domestic partnership	99 (16%)
Living apart together (LAT)	23 (4%)
Single	90 (15%)
Widow/widower	20 (3%)
Divorced	46 (7%)
Others	4 (1%)
Education	
Elementary	11 (2%)
Lower secondary	88 (14%)
Upper secondary	150 (24%)
Tertiary	363 (59%)
Others	6 (1%)
Working status (% currently working)	357 (58%)
Relative with dementia	276 (45%)
Non-relative with dementia	132 (21%)

^a^The numbers of participants (percentages) are shown unless otherwise stated

### Analysis of the psychometric characteristics of the MCLHB-DRR scale

#### Scale descriptives

The mean (SD) MCLHB-DRR subscale scores were 10.1 (2.7; range = 4 to 18) for perceived susceptibility, 13.9 (3.7; range = 5 to 25) for perceived severity, 12.6 (2.9; range = 4 to 20) for perceived benefits, 8.0 (2.5; range = 4 to 15) for perceived barriers, 10.2 (3.1; range = 4 to 19) for cues to action, 14.5 (2.3; range = 4 to 20) for general health motivation and 5.8 (1.7; range = 2 to 10) for self-efficacy. All subscale scores were approximately normally distributed. Item response scores of the MCLHB-DRR scale ranged from 1.9 (0.8; item 15) to 4.1 (0.7; item 24).

#### Exploratory factor analysis

EFA analysis with extraction method Maximum Likelihood and Oblimin rotation was used to assess the number of factors, because the data were roughly normally distributed. First, a seven factor solution was evaluated, as the original MCLHB-DRR scale consists of seven subscales. All items had an inter-item correlation greater than 0.20 with at least one of the other items. The correlation between item 1 and item 2 was 0.86 (*p* < 0.001), the correlation between item 1 and item 3 was 0.77 (p < 0.001) and the correlation between item 2 and item 3 was 0.82 (p < 0.001). Although these items had high inter-item correlations, they still measured something else (r < 0.90) and loaded on their intended factors. Therefore, none of these items was deleted. All other inter-item correlations did not exceed 0.70. The Bartlett’s test of sphericity was significant, indicating that the data was adequate for factor analysis (*p* < 0.001). The first seven factors had eigenvalues greater than 1.00. The eigenvalues and the cumulative percentages of explained variance of the first seven factors in brackets were 5.86 (21.7%), 2.94 (32.6%), 2.52 (41.9%), 2.10 (49.7%), 1.56 (55.5%), 1.23 (60.0%) and 1.03 (63.9%), respectively. The scree plot also suggested a seven factor model.

Almost all items loaded on their intended subscales and did not have any significant cross-loadings. However, item 10 did not have a factor loading greater than 0.30 on any of the factors. Therefore, item 10 was deleted. Item 26 had a significant cross-loading (cross-loading = 0.37) on the perceived benefits subscale. Furthermore, items 4, 5, 7, 9, 13, 25 and 26 had factor loadings between 0.30 and 0.50 on their intended factors (Table 2). Inclusion of these items was assessed in the next step by evaluating the internal consistency of their subscale. The inter-scale correlations between the subscale factors ranged from − 0.13 to 0.51.

**Table 2:** Exploratory factor analysis of the MCLHB-DRR scale (*N* = 618, Maximum Likelihood with Oblimin rotation)

		Factor 1	Factor 2	Factor 3	Factor 4	Factor 5	Factor 6	Factor 7
Q1	My chances of developing dementia are great	-0.02	**0.90**	0.00	-0.03	-0.03	0.03	0.04
Q2	I feel that my chances of developing dementia in the future are high	0.00	**0.97**	0.02	-0.04	-0.03	0.00	-0.00
Q3	There is a strong possibility that I will develop dementia	0.04	**0.86**	-0.03	0.01	0.02	-0.03	0.04
Q4	Within the next 10 years I will develop dementia	-0.04	**0.33**	0.07	0.25	0.07	-0.04	-0.12
Q5	The thought of dementia scares me	0.01	0.08	0.02	**0.49**	0.05	0.09	0.10
Q6	When I think about dementia my heart beats faster	-0.06	-0.00	0.10	**0.81**	0.01	-0.02	-0.04
Q7	My feelings about myself would change if I develop dementia	0.04	-0.01	-0.12	**0.43**	-0.01	0.03	0.10
Q8	When I think about dementia I feel nauseous	-0.03	-0.03	0.03	**0.80**	0.06	-0.05	-0.12
Q9	It would be more serious for me to develop dementia than if I developed other diseases	0.03	0.05	0.06	**0.45**	-0.04	0.00	-0.03
Q10	Information and advice from experts may give me something that I never thought of, and may reduce my chance of developing dementia	0.15	0.01	0.18	0.17	-0.09	0.01	**0.20**
Q11	Changing my lifestyle and health habits can help me reduce my chance of developing dementia	0.07	0.06	0.06	0.01	-0.04	-0.05	**0.77**
Q12	I have a lot to gain by changing my lifestyle and health behaviour	-0.03	0.03	0.08	0.01	0.05	-0.01	**0.77**
Q13	Adapting to a healthier lifestyle and behaviour would prevent dementia for me	0.13	-0.06	0.10	0.06	0.10	0.01	**0.38**
Q14	I am too busy to change my lifestyle and health habits	0.02	-0.03	0.00	-0.02	**0.61**	-0.05	-0.01
Q15	My financial situation does not allow me to change my lifestyle and behaviour	0.02	-0.02	0.06	0.05	**0.62**	0.05	-0.07
Q16	Family responsibilities make it hard for me to change my lifestyle and behaviour	-0.01	0.04	-0.09	-0.02	**0.78**	0.03	0.05
Q17	Changing lifestyle and behaviour interferes with my schedule	-0.02	-0.02	0.07	-0.00	**0.68**	-0.06	0.06
Q18	Being forgetful makes me think I have to change my lifestyle and behaviour	0.02	0.01	**0.68**	-0.02	0.05	0.01	-0.03
Q19	Having risk factor(s) for dementia makes me think I have to change my lifestyle and behaviour	0.01	0.03	**0.81**	-0.03	-0.01	0.01	0.04
Q20	Learning more about dementia from the media makes me think I have to change my lifestyle and behaviour	-0.01	-0.03	**0.71**	0.02	-0.03	0.03	0.17
Q21	Knowing family member(s) with dementia makes me think I have to change my lifestyle and behaviour	0.07	0.04	**0.64**	0.03	0.08	-0.02	0.00
Q22	Nothing is as important to me as good health	-0.03	-0.11	0.05	0.10	-0.08	**0.51**	-0.10
Q23	I often think about my health	0.00	-0.01	-0.03	-0.02	0.03	**0.85**	0.01
Q24	I think I have to pay attention to my own health	0.07	0.05	-0.05	-0.09	-0.01	**0.63**	-0.00
Q25	I am concerned about my health	-0.07	0.09	0.17	0.12	0.06	**0.32**	0.12
Q26	I am certain that I can change my lifestyle and behaviour so I can reduce the risk of developing dementia	**0.47**	0.03	0.16	-0.05	-0.03	0.03	**0.37**
Q27	I am able to make differences that will change the risk of developing dementia	**1.02**	-0.00	0.02	0.03	0.04	0.02	-0.07

The factor loadings greater than 0.30 are shown in bold. The boxes show the predicted subscales according to the results from Kim et al. 2014 [26]

#### Internal consistency

Item-total correlation analysis showed that all items were positively correlated with the total MCLHB-DRR scale score. Item-total correlations ranged from 0.15 to 0.67. The item-total correlations of item 14 (*r* = 0.28), item 22 (*r* = 0.15), item 23 (*r* = 0.26) and item 24 (*r*_s_ = 0.19) were lower than 0.30. All other items had an item-total correlation above 0.30. Cronbach’s alpha values were α = 0.86 for perceived susceptibility, α = 0.76 for perceived severity, α = 0.76 for perceived benefits, α = 0.77 for perceived barriers, α = 0.84 for cues to action, α = 0.64 for general health motivation and α = 0.81 for self-efficacy, all indicating good internal consistency. Cronbach’s alpha of the perceived susceptibility, perceived benefits and general health motivation subscales could be elevated by deleting an item. Items 4, 13 and 25 already had low factor loadings (factor loadings < 0.50) and were therefore eliminated. After deletion of these items, Cronbach’s alpha values were α = 0.93 for perceived susceptibility, α = 0.80 for perceived benefits and α = 0.69 for general health motivation. Cronbach’s alpha of all subscales could not be raised any further after deleting these items (Table 3).

**Table 3:** Internal consistency of the subscales

Subscale	Dutch MCLHB-DRR scale; ***N*** = 618				English MCLHB-DRR scale; ***N*** = 617			
	No. of items	Range of scores	Mean ± SD	α	No. of items	Range of scores	Mean ± SD	α
Perceived susceptibility	4	4–18	10.1 ± 2.7	0.86^b^	4	4–19	NK	0.86
Perceived severity	5	5–25	13.9 ± 3.7	0.76	5	5–25	NK	0.73
Perceived benefits	3^a^	3–15	9.1 ± 2.3	0.76^c^	4	4–20	NK	0.69
Perceived barriers	4	4–15	8.0 ± 2.5	0.77	4	4–20	NK	0.74
Cues to action	4	4–19	10.2 ± 3.1	0.84	4	4–20	NK	0.68
General health motivation	4	4–20	14.5 ± 2.3	0.64^d^	4	4–20	NK	0.61
Self-efficacy	2	2–10	5.8 ± 1.7	0.81	2	2–10	NK	0.66

^a^Item 10 is deleted

^b^Cronbach’s alpha elevated to 0.93 if item 4 was deleted

^c^Cronbach’s alpha elevated to 0.80 if item 13 was deleted

^d^Cronbach’s alpha elevated to 0.69 if item 25 was deleted

Abbreviations: *α* Cronbach’s alpha, *MCLHB-DRR* Motivation to Change Lifestyle and Health Behaviours for Dementia Risk Reduction, *NK* Not known

#### Confirmatory factor analysis

CFA with Maximum Likelihood method was conducted to explore the model fit of the MCLHB-DRR scale. A seven factor model including all 27 items (model 1) was evaluated with CFA. The fit indices were indicating a moderate fit (RMSEA = 0.053, CFI = 0.920, TLI = 0.907, χ^2^/df = 2.743). A seven factor model with 23 items (excluding items 4, 10, 13 and 25) (model 2) showed an excellent fit (RMSEA = 0.043, CFI = 0.960, TLI = 0.951, χ^2^/df = 2.130), indicating model 2 had a better fit to the data than model 1 (Table 4). The factor loadings of model 2 ranged from 0.395 to 0.978 and were all statistically significant (Table 5).

**Table 4:** Goodness of fit indexes of MCLHB-DRR models

	Dutch MCLHB-DRR scale (model 1)	Dutch MCLHB-DRR scale (model 2)	English MCLHB-DRR scale
RMSEA	0.053	0.043	0.047
CFI	0.920	0.960	0.920
TLI	0.907	0.951	NK
χ^2^/df	2.743	2.130	2.380

Model 1 represents a seven factor model including all 27 items; model 2 represents a seven factor model including 23 items (without items 4, 10, 13 and 25)

Abbreviations: *RMSEA* Root Mean Squared Error of Approximation, *CFI* Comparative Fit Index, *TLI* Tucker-Lewis Index, *MCLHB-DRR* Motivation to Change Lifestyle and Health Behaviours for Dementia Risk Reduction, *NK* Not known

**Table 5:** Confirmatory Factor Analysis report

Subscales	Item	Factor Loading
Perceived severity	Q1	0.896*
	Q2	0.953*
	Q3	0.858*
Perceived severity	Q5	0.573*
	Q6	0.842*
	Q7	0.395*
	Q8	0.753*
	Q9	0.487*
Perceived benefits	Q11	0.842*
	Q12	0.797*
Perceived barriers	Q14	0.627*
	Q15	0.617*
	Q16	0.734*
	Q17	0.719*
Cues to action	Q18	0.671*
	Q19	0.825*
	Q20	0.793*
	Q21	0.717*
General health motivation	Q22	0.562*
	Q23	0.815*
	Q24	0.612*
Self-efficacy	Q26	0.978*
	Q27	0.702*

Results are shown for model 2. *p < 0.001

## Discussion

We demonstrated that the Dutch version of the MCLHB-DRR scale, consisting of 23 items, is a valid instrument to measure the beliefs and attitudes towards lifestyle and health behavioural changes for dementia risk reduction in people aged between 30 and 80 years old. EFA showed that nearly all items loaded on their intended factors without cross-loadings. Cronbach’s alpha varied from 0.69 to 0.93, indicating good internal consistency. CFA confirmed that a seven factor model including 23 items (without items 4, 10, 13 and 25) had an excellent fit to the data (RMSEA = 0.043, CFI = 0.960, TLI = 0.951, χ^2^/df = 2.130).

Items 4, 10, 13 and 25 had low factor loadings and were therefore not included in the final Dutch version of the instrument. This could possibly be explained by differences in knowledge of dementia and dementia prevention between residents of Australia and the Netherlands. Australia is leading in the field of dementia prevention with the world first publicly-funded dementia prevention program [37]. This could have increased the public awareness about dementia and the prevention of dementia in Australia. In general, the Australian population scored higher on all subscales of the MCLHB-DRR scale, except for the self-efficacy subscale where the Dutch sample had a higher score [26]. Differences in cultural beliefs about general health, health behaviours and the prestige of health professionals may play a role. Another explanation is the age difference between the Australian and Dutch study populations [26]. The study population of the Australian study was 50 years and older whereas our population was between 30 and 80 years (73% was 50 years or older). People aged below 50 years might be less scared to develop dementia in the upcoming 10 years and might be less concerned about their health in comparison to people aged 50 years and over. However, our sensitivity analysis in which we only included people aged 50 years and over did not change our results in any way. Deficiencies in the translation process could be a third explanation. The translation of item 10 slightly changed, as the part of the sentence ‘may give me something that I never thought of’ is not included in the Dutch translation.

### Strengths and limitations

To our knowledge, this was the first study that validated the MCLHB-DRR scale in the Dutch general population. A major strength of the current study was the random sample, as the information letter was send to randomly selected residents of the municipality of Groningen. Another strength is the adequate sample size, consisting of a total number of 618 participants. Besides, we followed formal guidelines presented by Beaton et al. (2000) during the translation process [31].

This study also had certain limitations. The response rate of the current study was 14%, which is relatively low. However, we used several methods which have shown to increase the response rate to electronic surveys, such as a lottery to win a voucher, an offer to receive survey results on population level, a personalised invitation letter, an easily accessible link to the survey and a deadline to complete the survey [38, 39]. In our study, 59% of the participants completed tertiary education, which is higher than the percentage completing tertiary education in Dutch residents aged 45 years and over (26%) [40]. Therefore, the sample is not fully representative for the Dutch general population.

### Recommendations for future research

First, assessing the reliability and responsiveness of the Dutch MCLHB-DRR scale would be a valuable addition for future research. Second, a part of the study population might not be familiar with the health behaviours that decrease the risk of developing dementia. Future research could consider informing participants about these health behaviours before filling in the MCLHB-DRR scale. Further research should also examine the association between the motivation to change lifestyle and health behaviours for dementia risk reduction and actually conducting this behaviour in daily life.

### Implications

This scale can be useful in developing and evaluating interventions aimed at dementia risk reduction in various ways. Firstly, this instrument might help to predict people who will comply with an intervention program aimed at dementia prevention. Secondly, this instrument can be used in developing tailored interventions based on a person’s motivations and beliefs. For example, if an individual scores low on the perceived benefits subscale, it would be convenient to educate this individual about how changing lifestyle and health behaviours could reduce its risk of dementia. Finally, assessing the beliefs towards lifestyle and health behavioural changes in the community population of the Netherlands may help to develop media campaigns or education programs focused on dementia prevention.

## Conclusion

In summary, we have demonstrated that the translated and adapted Dutch version of the MCLHB-DRR scale, consisting of 23 items, is a valid instrument to assess health beliefs and attitudes towards dementia and dementia risk reduction in the Dutch general population aged between 30 and 80 years old. The MCLHB-DRR scale can be used in the development and evaluation of lifestyle interventions and media campaigns aimed at dementia risk reduction.

## Data Availability

The Dutch translation of the MCLHB-DRR scale and datasets analysed during the current study are available from the corresponding author on reasonable request.

## Abbreviations

MCLHB-DRR: Motivation to Change Lifestyle and Health Behaviours for Dementia Risk Reduction
EFA: Exploratory factor analysis
CFA: Confirmatory factor analysis
HBM: Health Belief Model
SD: Standard deviation
RMSEA: Root Mean Squared Error of Approximation
CFI: Comparative Fit Index
TLI: Tucker-Lewis Index
