# Correction to: Cross-cultural validation of the motivation to change lifestyle and health behaviours for dementia risk reduction scale in the Dutch general population

**DOI:** 10.1186/s12889-020-09229-9

**Published:** 2020-07-21

**Authors:** Tessa Joxhorst, Joyce Vrijsen, Jacobien Niebuur, Nynke Smidt

**Affiliations:** Department of Epidemiology, University of Groningen, University Medical Centre Groningen, Hanzeplein 1, FA40, PO Box 30 001, 9700 RB Groningen, The Netherlands

**Correction to: BMC Public Health (2020) 20:788**

**https://doi.org/10.1186/s12889-020-08737-y**

It was highlighted that the original article [1] contained an error in the legend of Table 2. This Correction article shows the correct Table 2 and legend. The original article has been updated.

**Table 2:** Exploratory factor analysis of the MCLHB-DRR scale (*N* = 618, Maximum Likelihood with Oblimin rotation)

	Factor 1	Factor 2	Factor 3	Factor 4	Factor 5	Factor 6	Factor 7
Q1 My chances of developing dementia are great	− 0.02	**0.90**	0.00	−0.03	−0.03	0.03	0.04
Q2 I feel that my chances of developing dementia in the future are high	0.00	**0.97**	0.02	− 0.04	− 0.03	0.00	− 0.00
Q3 There is a strong possibility that I will develop dementia	0.04	**0.86**	− 0.03	0.01	0.02	− 0.03	0.04
Q4 Within the next 10 years I will develop dementia	− 0.04	**0.33**	0.07	0.25	0.07	− 0.04	− 0.12
Q5 The thought of dementia scares me	0.01	0.08	0.02	**0.49**	0.05	0.09	0.10
Q6 When I think about dementia my heart beats faster	− 0.06	− 0.00	0.10	**0.81**	0.01	− 0.02	− 0.04
Q7 My feelings about myself would change if I develop dementia	0.04	− 0.01	− 0.12	**0.43**	− 0.01	0.03	0.10
Q8 When I think about dementia I feel nauseous	− 0.03	− 0.03	0.03	**0.80**	0.06	− 0.05	− 0.12
Q9 It would be more serious for me to develop dementia than if I developed other diseases	0.03	0.05	0.06	**0.45**	− 0.04	0.00	− 0.03
Q10 Information and advice from experts may give me something that I never thought of, and may reduce my chance of developing dementia	0.15	0.01	0.18	0.17	−0.09	0.01	0.20
Q11 Changing my lifestyle and health habits can help me reduce my chance of developing dementia	0.07	0.06	0.06	0.01	− 0.04	− 0.05	**0.77**
Q12 I have a lot to gain by changing my lifestyle and health behaviour	− 0.03	0.03	0.08	0.01	0.05	− 0.01	**0.77**
Q13 Adapting to a healthier lifestyle and behaviour would prevent dementia for me	0.13	− 0.06	0.10	0.06	0.10	0.01	**0.38**
Q14 I am too busy to change my lifestyle and health habits	0.02	− 0.03	0.00	− 0.02	**0.61**	−0.05	−0.01
Q15 My financial situation does not allow me to change my lifestyle and behaviour	0.02	− 0.02	0.06	0.05	**0.62**	0.05	− 0.07
Q16 Family responsibilities make it hard for me to change my lifestyle and behaviour	− 0.01	0.04	− 0.09	− 0.02	**0.78**	0.03	0.05
Q17 Changing lifestyle and behaviour interferes with my schedule	− 0.02	− 0.02	0.07	− 0.00	**0.68**	− 0.06	0.06
Q18 Being forgetful makes me think I have to change my lifestyle and behaviour	0.02	0.01	**0.68**	− 0.02	0.05	0.01	− 0.03
Q19 Having risk factor(s) for dementia makes me think I have to change my lifestyle and behaviour	0.01	0.03	**0.81**	− 0.03	− 0.01	0.01	0.04
Q20 Learning more about dementia from the media makes me think I have to change my lifestyle and behaviour	− 0.01	− 0.03	**0.71**	0.02	− 0.03	0.03	0.17
Q21 Knowing family member(s) with dementia makes me think I have to change my lifestyle and behaviour	0.07	0.04	**0.64**	0.03	0.08	− 0.02	0.00
Q22 Nothing is as important to me as good health	−0.03	−0.11	0.05	0.10	−0.08	**0.51**	−0.10
Q23 I often think about my health	0.00	− 0.01	− 0.03	− 0.02	0.03	**0.85**	0.01
Q24 I think I have to pay attention to my own health	0.07	0.05	− 0.05	− 0.09	− 0.01	**0.63**	− 0.00
Q25 I am concerned about my health	− 0.07	0.09	0.17	0.12	0.06	**0.32**	0.12
Q26 I am certain that I can change my lifestyle and behaviour so I can reduce the risk of developing dementia	**0.47**	0.03	0.16	− 0.05	−0.03	0.03	**0.37**
Q27 I am able to make differences that will change the risk of developing dementia	**1.02**	− 0.00	0.02	0.03	0.04	0.02	−0.07

The factor loadings greater than 0.30 are shown in bold
